# Safety of endoscopic N-Butyl-2 Cyanoacrylate injection for the treatment of bleeding gastric varices in children

**DOI:** 10.12669/pjms.346.16044

**Published:** 2018

**Authors:** Lubna Kamani, Baseer Sultan Ahmad, Muhammad Arshad, Pervez Ashraf

**Affiliations:** 1 *Dr. Lubna Kamani, FCPS, MRCP(UK), FRCP, Department of Gastroenterology and Hepatology, Liaquat National Hospital &Medical College, Karachi, Pakistan*; 2 *Dr. Baseer Sultan Ahmad, FCPS, Department of Gastroenterology and Hepatology, Liaquat National Hospital &Medical College, Karachi, Pakistan*; 3 *Dr. Muhammad Arshad, FRCS, Department of Pediatric Surgery, Liaquat National Hospital &Medical College, Karachi, Pakistan*; 4 *Dr. Pervez Ashraf, MRCP, FRCP, Department of Gastroenterology and Hepatology, Liaquat National Hospital &Medical College, Karachi, Pakistan*

**Keywords:** Cirrhosis, Extra hepatic portal venous obstruction (EHPVO), Fundal varix obliteration (FVO), Portal hypertension

## Abstract

**Objective:**

To determine the safety and efficacy of N-butyl 2-cyanoacrylate in bleeding gastric varices in children.

**Methods:**

This retrospective observational study was conducted in the Department of Gastroenterology and Pediatric Surgery in Liaquat National Hospital Karachi between January 2010 and January 2017. Gastric fundal varices were obliterated in pediatric population with single shot of N-butyl-2 Cyanoacrylate 0.50ml diluted with 0.50ml of Lipoidal with use of forward-viewing video endoscope with 22-gauge needle. The primary outcome was primary hemostasis, Secondary outcome was complications, re-bleeding and mortality.

**Results:**

Thirty patients was included in the study, 18(60%) were male with mean age of 7.12± 2.9 years. Non cirrhotic portal hypertension was the most common etiology in 15 (50%) patients, followed by liver cirrhosis secondary to hepatitis B and D co-infection in 6(20%) patients. Primary hemostasis was achieved in 29 (96.7%), while 3(10%) patients developed re-bleeding after 48 hours, and hemostasis was achieved after second session of endoscopic obliteration. Abdominal pain and fever developed in 3(10%) patients which was managed conservatively. Mortality was observed in 1(3%) of cases due to sepsis after shunt surgery.

**Conclusion:**

Endoscopic fundal varix obliteration with N Butyl-2 cyanoacrylate was safe and effective in treatment of gastric variceal hemorrhage in children.

## INTRODUCTION

Portal hypertension is a pathological entity which indicates elevated pressures in the hepatic portal system. It is an outcome of increased vascular resistance or increased blood flow in portal system, it is technically stated in terms of portal pressure gradient or a hepatic venous pressure gradient (HVPG) equal to or greater than 5 mmHg in children.^1^ In pediatric population extra hepatic portal venous obstruction (EHPVO) remains the most dominating cause of portal hypertension in non-urban population whereas liver cirrhosis in industrialized population.^2^-^4^ HVPG greater than 10 mmHg results in complications such as variceal hemorrhage, ascites, portosystemic encephalopathy, thrombocytopenia, splenomegaly and pulmonary complications.^5^

Acute variceal hemorrhage is a catastrophic and fatal event in children resulting from the rupture of esophageal and gastric varices (GV), which stipulate urgent intervention. An adequate and safe approach in managing esophageal varices is endoscopic variceal ligation (EVL), it is highly recommended and standard of care first line treatment in the management in both adults and children.^6^,^7^ GV are notorious and bleed massively, the severity of bleeding is more intense and life-threatening.^8^ Management of gastric variceal hemorrhage is a challenging task and requires expertise to treat since it can re-bleed aggressively with high mortality and morbidity rates.^9^

Different treatment modalities for treating gastric variceal hemorrhage in adults include endoscopic gastric varix obliteration with directly injecting N-butyl-2-cyanoacrylate into the varix, Transjugular Intrahepatic Portosystemic Shunt (TIPS) and balloon-occluded retrograde trans venous obliteration (B-RTO) requiring skillful radiological expertise and finally portosystemic shunt surgeries. The Baveno VI consensus guidelines propose the use of N-butyl-2-cyanoacrylate (histoacryl) as a treatment of choice in endoscopic obliteration of gastric varix in adults.^10^

The Baveno V consensus statement on portal hypertension in children suggest the use of N-butyl-2-cyanoacrylate (histoacryl) for isolated gastric varices and gastro esophageal varices Type-II (GOV2) but there are no definite recommendations, as it requires more controlled trials for validation.^11^

Historically Soehendrain in 1986 proposed for the first time, the use of N-butyl 2-cyanoacrylate, a tissue adhesive agent, which was endoscopically injected into the bleeding gastric varices.^12^ Systemic embolism, is one of the fatal complications seen in patients treated with cyanoacrylate, though the incidence is between 0.5 to 4.3% in adults.^13^,^14^

N-butyl, 2-cyanoacrylate is glue like substance with adhesive properties, it induces the process of polymerization and solidifies when in contact with water or blood promoting obliteration of the vessel and also promotes local thrombosis. The American Society of gastrointestinal endoscopy guidelines on tissue adhesives recommend the mixing of cyanoacrylate with lipoidal with a standard ratio of 1:1 up to 1:1.6 in adults. Appropriate dilution delays fast polymerization of cyanoacrylate and allows adequate endoscopic administration with injector and prevents its adherence to catheters and endoscopes. Data on safety and efficacy of N-butyl-2-cyanoacrylate in managing bleeding gastric varices in children is scarce.^15^

Our main objective was to observe the safety and effectiveness of N-butyl 2-cyanoacrylate injection in bleeding gastric varices in pediatric population and also observe its outcomes which include achievement of hemostasis, re-bleeding and complications.

## METHODS

The study was conducted in Department of Gastroenterology and Pediatric Surgery at Liaquat National Hospital, Karachi Pakistan. Patients medical record were reviewed extensively from January 2010 to January 2017after receiving an approval from the institution`s ethical review board.

Pediatric population up to 14 years of age manifesting acute upper gastrointestinal bleeding or malena as a consequence from the rupture of GV were included for study analysis. We included patients of both liver cirrhosis and EHPVO. Diagnosis was made on the basis of laboratory investigations complete blood count liver function tests, prothombin time, albumin, ultrasonography of liver and abdomen, computerized tomography (CT) scan abdomen and endoscopic findings and in some cases liver biopsy was also done. EHPVO was confirmed with contrast enhanced CT angiography.

The patients presenting with malena and /or hematemesis were admitted in high dependency unit for resuscitation. Hemodynamic stability was achieved with intravenous fluids, antibiotics, andoctreotide1 microgram/kg bolus dose and then 1 microgram/kg/hour in continuous infusion for at least 3 days with transfusion of packed red blood cells if required.

Endoscopy was performed after informed consent within 24 hours of hemodynamic stability. Endoscopic criteria to label hemorrhage from GV was integrated, if anyone of the following was present:

1. Spurting or oozing of fresh blood from varix
2. Presence of blood clot or ulcer on varix
3. Occurrence of discrete large varix with no other source of hemorrhage and with no evidence of esophageal varices.^8^

We used Sarin classification to classify gastric varices. Gastro-esophageal varices Type-1 (GOV1)is extension of esophageal varices along the lesser curve of the stomach, gastro-esophageal varices Type-2 (GOV2) is continuation of EV to the gastric fundus along greater curvature of stomach, isolated gastric varices Type-1 (IGV1) are present in fundus of stomach and lastly isolated gastric varices Type-2 (IGV2), are also labeled ectopic varices, they can be present anywhere in the pylorus antrum, or corpus, of stomach.^13^ We used forward viewing video endoscope (Olympus Optical Corporation, Tokyo, Japan) for endoscopic varix obliteration. Priming of sclerotherapy needle of 22-gauge with 0.5 ml lipiodal was to ensure the dead space was filled. For prevention of embolization and very early polymerization equal amount of lipiodal and N-butyl, 2-cyanoacrylate (histoacryl) with ratio (1:1) was mixed and endoscopically injected into the varix (Figure A&B). An x-ray was done at the end of procedure. Distilled water was flushed just before after each injection. The glue was used with caution to prevent equipment damage and endoscopist eyes by using goggles.

**Fig.1:**
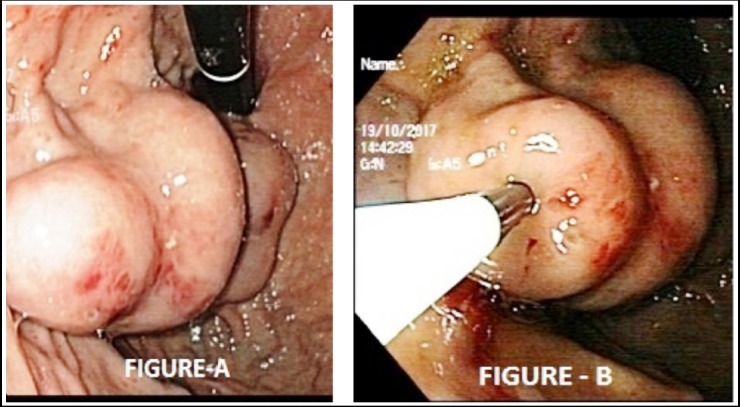
Gastric Varix with overlying ulcer seen in Fig.1A where as in Fig.1B Gastric Varix obliterated with endoscopic injection of N-butyl 2-cyanoacrylate.

Primary homeostasis is defined as endoscopic homeostasis, with normalization of vital signs and no hemoglobin drop or re-bleeding within 48 hours of gastric varix obliteration. Early re-bleeding is defined as hypotension or 2gm /dl drop in hemoglobin from base line requiring blood transfusion or history of hematemesis or malena within 48 hours of gastric varix obliteration whereas late re-bleeding was defined as recurrence of bleeding after 48 hours up to 30 days.

Technical failure is defined as failure to achieve hemostasis or stop bleeding from the varix during endoscopy. After discharge all patients were closely monitored in outpatient department for 24 months, till the time they went for liver transplant or shunt surgery.

## RESULTS

Thirty patients were enrolled in this study which were managed as acute gastric variceal hemorrhage. The mean age was 7.12± 2.9 years. Out of 30 patients 18(60%) were males. Non-cirrhotic portal hypertension which included extra hepatic portal vein obstruction was the most common etiology of portal hypertension comprising 15(50%) patients and other causes illustrated in (Table-I). Majority of the patients presented with malena 17(56.7%) whereas hematemesis was present in 5(16.7%) children whereas rest of the patients 8(26.7%) had both malena and hematemesis.

**Table-I:** Etiology of portal hypertension.

Etiology	No. of patients
EHPVO	15(50%)
Cirrhosis (HBV&HDV)	6 (20%)
Biliary Atresia	4(13.3%)
Wilson Disease	1(3.3%)
Budd CHiARI Syndrome	1(3.3%)
CHF	1(3.3%)
Autoimmune Hepatitis	1(3.3%)
Familial Intrahepatic Cholestasis(PFIC)	1(3.3%)

The mean Pediatric End-stage Liver Disease (PELD) score was 7.81 ±1.28 in cirrhotic patients. Among children, 26 (86.7%) experienced primary gastric variceal hemorrhage (hemorrhage from rupturing of gastric varices for the first time) whereas 4 (13.3%) patients encountered secondary gastric variceal hemorrhage (previous history of hemorrhage from ruptured esophageal varices). Urgent need of blood transfusion was considered necessary in 19(63.3%) patients whereas 11(37.7%) did not require any blood products.

Most common type of gastric varices on endoscopic findings was isolated gastric varix Type-1 (IGV1) in 18(60.0%) children and gastro esophageal varices Type-2 (GOV2) in 12(40%) patients. About 12(40%) of patients underwent both EVL and FVO.

Technical success and primary homeostasis was accomplished in 29(96.7%) patient. One (3.3%) patient had ongoing hemorrhage which was not controlled endoscopically so the patient was referred for emergent shunt surgery who later died due to sepsis.

Rebleeding was observed in 3(10.0%) patients after 48 hours and required second session obliteration with of N-butyl, 2-cyanoacrylate. No major complications were observed in patients, only 3(10%) patient developed fever and abdominal pain and managed conservatively.

The patients with liver cirrhosis and bilary atresia 4(13.3%) were followed and referred to liver transplant centre. Out of 15(50%) patients with extra hepatic portal vein obstruction (EHPVO) 9(29.7%) patients underwent distal splenorenal shunt surgery, while 2(6.6%) patients were lost to follow-up. The other three patients had complex hepatoportal anatomies who did not meet criteria for shunt surgery. They were kept on secondary prophylaxis with non-selective beta blockers. Mean follow-up of these patients were 12.66±0.44 months.

## DISCUSSION

Portal hypertension and its complications are difficult to manage, as gastric variceal hemorrhage is life threatening and options are limited in pediatric population. Gastric varices are mostly prevalent in patients with portal hypertension particularly with extra hepatic portal venous thrombosis (EHPVO) in children.^16^ Our study is a retrospective analysis. It also established similar results with as 15(50%) of them were diagnosed as extra hepatic portal venous obstruction (EHPVO).

Sarin et al has also concluded that patients with EHPVO frequently had gastric varices as compared with patients with cirrhosis (31% *vs*. 17%, *P*<0.01)and isolated gastric varix (IGV1) have intense risk to bleed as compared to gastro esophageal varix (GOV) in adults.^13^ In another study comprising 274 EPHVO patients, highlighted the fact that once esophageal varices are obliterated with band ligation, the gastric varices IGV1 becomes more prevalent (From 1% to 14%, *P*<0.001) and are more likely to bleed.^17^ In our study gastric varix (IGV1) was dominantly seen in18(60%) of patients and GOV2 in 12(40%) patients. Both endoscopic variceal ligation and fundal varix obliteration was done in 12(40%) patients.

In published studies fever and abdominal pain are the most frequent complains after cyanoacrylate injection reported in 90% of patients which resolves spontaneously with antibiotics and analgesics.^16^ Systemic embolization is a devastating complication, which is a consequence of over dilution resulting in cerebral embolization causing cerebral strokes, pulmonary embolism, portal vein thrombosis, and splenic infarction reported up to 4.3% in patients, other complications includes bleeding ulcers, sepsis accounting 3.3% and 1.3% of patients in adults.^17^

In our study, fortunately none of our patients had life threatening complications; only 3(10%) patients had fever and abdominal pain post procedure conservatively managed with analgesics and broad spectrum antibiotics resolved spontaneously.

Special safety precautions should be undertaken by the endoscopy technicians and the endoscopist to protect eyes and endoscopy biopsy channel. Huang et al. revealed damage to the endoscopy channel after the use of histoacryl and underwent costly repair. In our study, we strictly followed the protocol and no endoscopy equipment was damaged and endoscopist conducted the procedure without complications.^18^ N-butyl, 2-cyanoacrylate had initially controversial safety due to immediate and late complications, as some complications were fatal.^19^,^20^

In adults the volume of N-butyl, 2-cyanoacrylate injected into the varix varies between 0.5 to 1ml as by experts, but further studies are needed to validate ideal volume to obliterate varix. Some experts suggest volume can be modified to the size of varix.^19^-^21^ Oh et al. elaborated their experience in their study of 21 pediatric patients, the mean volume to obliterate in gastric varices ranged from 0.25 to 0.5 ml with no complication.^22^ Our study also revealed similar results, the mean volume used were 0.26±0.10ml.

Primary hemostasis was achieved in 96.7% of patients in our study, these results are quite comparable to results in adult population.^23^,^24^ Similarly Rivet et al conducted a study among infants demonstrated primary hemostasis achieved up to 100%.^25^

Rebleeding is an established complication accounting 0.1 to 6.3% in adults.^26^,^27^ Rebleeding occurs due to necrosis and ulceration at the sclerotherapy site, or injecting hisotacryl at extra variceal site or late bleeding consequence from incomplete obliteration or extrusion of glue from the varix. Podder et al in his recent published research in management of gastric varices in non-cirrhotic portal hypertension in children revealed rebleeding rate of 14% and mortality of 9%.^28^ In our study we reported rebleeding rate of 10% with mortality rate of 3.3%.

In adult population the severity of gastric variceal hemorrhage is more intense, with increase chances of rebleeding (25%-55%) and with significantly higher fatality rate (26%-89%) as compared to pediatric population(0%-8%), and it needs further validation.^28^,^29^

Baveno VI pediatric satellite symposium for portal hypertension recommended transjugular intrahepatic portosystemic shunt (TIPS) procedure and portosystemic shunt surgery (MesoRex) for primary and secondary prophylaxis of variceal bleeding.^30^ Transjugular intrahepatic portosystemic shunt (TIPS) and shunt surgery are effective and safe.^31^,^32^ These procedures require specialized centers with trained interventional radiologist and pediatric vascular surgeons and this facility is not available in most centers in Pakistan. Liver transplantation is the ultimate treatment with patients with cirrhosis.

## CONCLUSION

Our study proposes endoscopic fundal varix obliteration using N-butyl-2-cyanoacrylate is safe and effective in children for the initial hemostasis from bleeding gastric varices especially in center’s where emergency shunt surgery service and transjugular intrahepatic portosystemic shunt (TIPS) is not available.

### Authors’ Contribution

**LK** conceived, designed, editing and is responsible for integrity of the study.

**BSA** did data collection and manuscript writing, statistical analysis, integrity of the research work.

**MA and PA** did review and final approval of manuscript to be published.
